# Clinical Manifestations’ Spectrum of Smartphone Addiction: Moving from an Addiction toward a Clinical Syndrome

**DOI:** 10.2174/0117450179295575240520064919

**Published:** 2024-06-07

**Authors:** Mudar Alwazzeh, Muhdammad Harfouch, Manal Ahmed Hasan, Safi Alqatari, Abir Hamad AlSaid, Marwan Jabr Alwazzeh

**Affiliations:** 1 Department of Internal Medicine, Faculty of Medicine, Al Andalus University for Medical Sciences, Tartus, Syria; 2 Division of Rheumatology, Department of Internal Medicine, Imam Abdulrahman Bin Faisal University, King Fahad Hospital of the University, Al-Khobar, Saudi Arabia; 3 Division of Respiratory Medicine, Department of Internal Medicine, Faculty of Medicine, Imam Abdulrahman Bin Faisal University, King Fahad Hospital of the University, Al-Khobar, Saudi Arabia; 4 Division of Infectious Diseases, Department of Internal Medicine, Faculty of Medicine, Imam Abdulrahman Bin Faisal University, King Fahad Hospital of the University, Al-Khobar, Saudi Arabia

**Keywords:** Smartphone addiction, Distress, Pain, Depression, Anxiety, Sleep disorders, Smart device syndrome

## Abstract

**Background:**

Smartphone addiction is an emerging type of addiction in the digital era, characterized by smartphone dependence that negatively affects human health with a wide range of psychological and physical manifestations.

**Objective:**

This study aimed to evaluate the detailed clinical manifestations of smartphone addiction as a delineated clinical syndrome.

**Methods:**

A cross-sectional study design was employed to assess smartphone addiction prevalence and its health impacts among Syrian undergraduates using the Smartphone Addiction Scale-Short Version (SAS-SV 2013), the Kessler psychological distress scale (K-6), and a comprehensive assessment of the clinical manifestations frequently linked to smartphone addiction in the literature. Different statistical modeling techniques were applied; a *P* value of < .05 was considered statistically significant.

**Results:**

Of 1532 invited undergraduates, 1401 (91.45%) completed the assessment adequately. Most participants were females (59.7%) and below 23 years of age (73.2%). The prevalence of smartphone addiction was 67.80%; statistically significant smartphone addiction associations were revealed with psychological distress (*P* < .0001) with odds ratios of 3.308. Most screened physical manifestations also showed a significant association with smartphone addiction.

**Conclusion:**

A high prevalence of smartphone addiction was observed with a broad spectrum of associated mental and physical manifestations. As smart device addiction becomes a global health concern, combining the clinical findings reported in the related literature into one clinical identity is necessary to develop a holistic management approach for the delineated clinical syndrome.

## 1 INTRODUCTION

Smartphone Addiction (SA) is a relatively new behavioral addiction that has emerged in the last few decades, along with other digital addictions, such as internet and gaming addiction. It is also called problematic smartphone use, overuse, or excessive smartphone use. However, the SA term has been used more frequently in the literature to describe the uncontrolled use of smartphones with tolerance and withdrawal symptoms regardless of psychological, physical, and social harmful consequences [1-3]. It has been estimated that the number of smartphone subscriptions worldwide would increase to 6.8 billion by 2023, meaning smartphones to be in the hands of around 85% of the global population [4]. Furthermore, the time spent on these devices is rising due to the widespread and increased affordability, online jobs, smart applications, and easy portability, which could, in turn, increase the number of SA patients worldwide.

Research on SA and its health outcomes has shown significant mental health consequences and a consistent association with anxiety and depression [5-7]. In addition, SA has been linked to a higher risk of suicidal ideation and attempts [8]. Moreover, SA has been reported to be associated with poor sleep quality and daytime sleepiness [5, 6]. Notably, high SA rates have been reported to be commonly found in adolescents and young people, especially undergraduate students, with adverse effects on physical activity, communication skills, and academic performance [9, 10]. Finally, the relationship between SA and psychiatric disorders seems to be overlapping; however, the role of already existing psychiatric disorders as risk factors for developing SA has not been widely investigated yet.

In terms of physical health consequences, a significant correlation was observed between SA and musculoskeletal complaints, including neck, back, wrist, thumb, hips, and feet [5, 11]. Other health problems linked to SA include an increased probability of psoriatic arthritis, dry eye disease, functional gastrointestinal disorder, eating disorders, and accident risk [12-15]. Furthermore, cervical disc degeneration and structural changes with gray matter abnormalities in the brain have been reported [16, 17].

Given the fact that previous research has shown a significant association between SA and musculoskeletal pain in different body areas (neck, shoulders, arms, wrists, back, *etc*.) along with mental and physical manifestations (fatigability, headache, depression, sleep disorder, abdominal pain, *etc*.), this study aimed to investigate SA’s association with the psychological distress and a wide range of physical manifestations linked to SA, and to approach such disorder as a delineated clinical syndrome.

## 2 MATERIALS AND METHODS

### 2.1 Setting and Participants

**Fig. (1):**
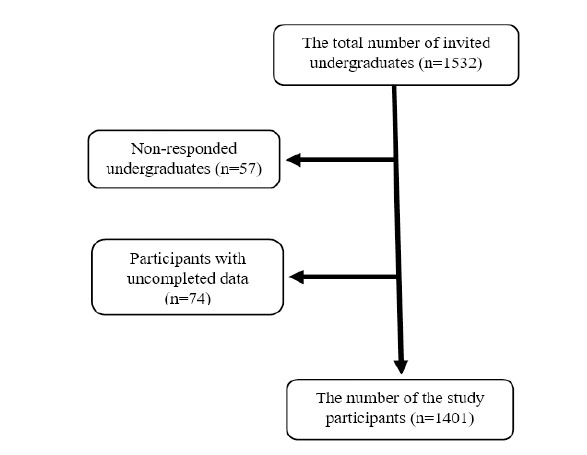
Smartphone addiction **s**tudy’s flow chart.

Between 1^st^ April and 31^st^ May, 2022, 1532 undergraduates from all Syrian higher education institutions were invited to be included in this cross-sectional study through convenience sampling. All participants were 18 years of age or older; of the 1532 invited students, 1401 (91.45%) had responded and completed the assessment adequately. One hundred and thirty-one (8.55%) participants were excluded because they did not respond or because of a lack of assessment information (Fig. **1**). The estimated required sample size was 825 based on the mean prevalence of SA reported in previous studies, with a sample size ratio (SA non-addicted/addicted group) of 1.57 and a two-side significance level of 0.05.

### 2.2 Study Tools and Procedures

The relevant data were collected using a self-administered questionnaire including demographic variables, such as age, gender, marital status, number of family members, student’s major, academic year, and economic status. In addition, the study assessed three clinical dimensions: the diagnosis of SA, the presence of psychological distress, and a review of a set of clinical manifestations linked to SA in the literature.

Initially, informed consent was obtained after demonstrating the study’s objectives; no personal identification information was collected, and data security was ensured. The designed questionnaire was divided into five parts: (a) sociodemographic data; (b) smartphone use data: date of smartphone ownership, mean daily hours of usage, and purposes of using a smartphone; (c) smartphone addiction scale: a validated Arabic smartphone addiction scale-short version for adults (SAS-SV 2013) was used [18, 19]. This scale consists of 10 questions derived from The Diagnostic and Statistical Manual of Mental Disorders, fifth edition (DSM-5) criteria of substance addictions; these questions cover smartphone usage more than intended, inability to quit, craving or urge to use, needing more usage, presence of withdrawal symptoms, neglecting responsibilities, continuous usage despite adverse effects on health or social relationships, and the usage in risky situations. A Likert scale (ranging from 1 strongly disagree to 6 strongly agree) was applied to answer the scale questions (Appendix **1**). A positive smartphone addiction score of ≥33 for females and ≥31 for males, with 87.5% sensitivity and 88.6% specificity, was accepted [18]. (d) The Kessler psychological distress scale (K-6) for nonspecific psychological distress (Appendix **2**) was applied using an Arabic-validated version to evaluate the possibility of psychological distress in the last 30 days [20, 21]. This scale consists of 6 items that indicate mood or anxiety disorders, including feelings of nervousness, restlessness, hopelessness, and worthless-ness, the feeling that nothing can cheer up, and that doing everything is an effort. The reply of each item was obtained using a 5-level response scale, ranging from 0 to 4 (0=none of the time, 1= a little of the time, 2=some of the time, 3= most of the time, 4= all the time). The results were then calculated using a scoring scale ranging from 0 to 24; a score of ≥ 13 points demonstrated the presence of psychological distress. (e) To evaluate the clinical manifestations associated with SA, a set of clinical presentations was evaluated, including headache, fatigability, depression, sleep disturbance, and pain complaints in different body areas (jaw, shoulder girdle, upper arm, lower arm, hip, upper leg, lower leg, neck, upper back, lower back, chest, and abdomen).

### 2.3 Statistical Analysis

Data were imported after cleaning and presented using a Microsoft Excel sheet, and Statistical Product and Service Solutions (SPSS) version 26.0, IBM, USA, was used for statistical analysis. Categorical variables have been presented as frequency and percentage, and numerical variables as mean ± standard deviation; the normality of data was evaluated using the Shapiro-Wilk test. The researchers have applied different statistical modeling techniques, such as frequency distributions, graphic visualization, correlation tests, binary logistic regression, Relative Risk (RR), Odds Ratio (OR), and prevalence analyses. The chi-square test was used to assess the effects of SA on the development of psychological distress and other clinical manifestations; a *P*-value of < .05 was considered statistically significant. In addition, If the chi-square test was significant, the Mantel-Haenszel test was used to calculate the odds ratio and estimate the effect sizes.

## 3 RESULTS

### 3.1 Demographic Data

Of 1532 invited participants, 1401 (91.45%) completed the assessment adequately. Male and female participants were 565 (40.3%) and 836 (59.7%). In addition, 42.5% were 21-23 years old, and 69.5% were from a family with > 5 members. Most of the participants were single and had average economic status (Table **1**).

### 3.2 Smartphone Data

#### 3.2.1 Smartphone Use Data

Regarding smartphone usage data, most participants (52.0%) have owned smartphones for 5–8 years. Moreover, 50% of them used their smartphones on average 4–10 hours per day; the average daily screen time was 8.50 hours among smartphone-addicted participants and 6.76 hours among non-addicted participants. The mean purposes for smartphone use were social communication (90.2%) and internet surfing (80.9%) (Table **2**).

#### 3.2.2 Smartphone Addiction

Out of 1401 participants, 950 (67.80%) had SA according to the SAS-SV score (score sum of ≥33 for females and ≥31 for males), and the prevalence of smartphone addiction was 70.3% and 66.1% for male and female participants, respectively. The males were more likely to be smartphone-addicted than females (OR = 1.21). Moreover, families with five or more family members were more likely to have smartphone-addicted individuals than families with < 5 members (OR = 1.16). The binary logistic regression model was used to evaluate the association of participant age, mean daily screen time, and study major with SA (Table **3**). The participants of younger age groups were more likely to be smartphone-addicted than those ≥ 30 years of age (for the 18-20 age group, OR = 2.264; P = .011, and for the 21-23 age group, OR = 2.104; P = .018). In addition, the model showed a higher mean daily time of smartphone use as associated with an increase in the prevalence of SA. Similarly, sciences, economics, and medical sciences students were more likely to be smartphone-addicted (OR: 2.178, 2.035, and 2.170, respectively) with P < .05.

### 3.3 Psychological Distress and SA

**Fig. (2):**
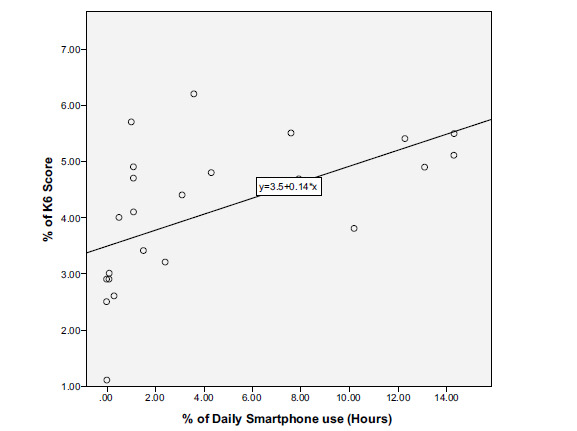
The correlation between the increase in daily smartphone use and K-6 score.

Regarding psychological distress, 559 (40.0%) of participants had a K-6 score of ≥ 13, indicating the presence of psychological distress, and 467 (82.1%) of them were smartphone addicts (P < .0001) with an estimated OR of 3.308. Moreover, the increase in daily smartphone use was correlated with an increase in K-6 scores (Fig. **2**).

### 3.4 Physical Manifestations and SA

Screening of physical health problems showed a strong association between SA and complaints of pain in different body areas (neck, upper back, lower back, abdomen, shoulder girdle, upper arm, lower arm, R. jaw, R. hip, R. upper leg, and R. lower leg). Moreover, SA was significantly associated with headache, fatigability, sleep disturbance, cognitive symptoms (impaired memory, concentration difficulties, or bradyphrenia), and depression (Table **4**).

## 4 DISCUSSION

The global spread of smart devices and their problematic overuse may lead to increased adverse health effects and be associated with emerging illnesses that are not well investigated yet. In this study, for the first time, we have shed light on SA among Syrian undergraduates and investigated SA's association with psychological distress and different physical complaints. In addition, the uniqueness of this study is that it has investigated the possibility of combining the linked SA clinical presentations into one clinical identity to develop a holistic management approach for the delineated clinical syndrome.

The prevalence of SA among the study population has been found to be 67.8%, aligning with the other studies conducted using SAS-SA [10, 23]. However, SA prevalence among Syrian undergraduates has been found to be much higher compared to the findings of other studies from Serbia (19.5%), Malaysia (47.9%), Turkey (46.9%), Lebanon (46.9%), and Brazil (33.1%) [24-28]. The high prevalence of SA among Syrian undergraduates might be explained by different factors, such as culture, personality traits, and other social determinants, such as disastrous circumstances in this country and its related stress burden.

The risk of SA has been notably correlated with a steady increase in the daily screen time of participants, being in agreement with previously performed studies [29, 30]. However, self-reporting of screen time might be affected by recall bias and other external factors and may not reflect actual screen use [31]. The male participants in this study were more likely to be smartphone-addicted than females; similar findings have been observed by Chen *et al*., Davey *et al*., and Choi *et al*. [32-34]. In contrast, being female has been reported as a risk factor for SA in other studies conducted in different parts of the world [35-37]. Moreover, some studies have found no association between SA and gender [10, 38]. The age, social characteristics, and differences between studied populations can explain these heterogeneous findings of the association between gender and SA.

Regarding the association between age and SA, the binary logistic regression analysis showed the participants of younger age groups to be more likely to be smartphone-addicted than those older, which is consistent with previous studies that have identified adolescents and young adults as risk groups for SA [36, 39-42]. The suggested reasons behind the increased SA rate include high social media use, peer pressure, lack of self-control, escapism from reality, and social phobia [41, 43-47]. The primary purposes for smartphone use among the study population have been reported to be social communication and internet surfing, suggesting social media use as a contributing factor to SA development.

In line with previous studies, families with five or more family members were more likely to have smartphone-addicted individuals than families with < 5 members [23, 48]. Multiple familial factors may increase the probability of SA in big families, such as lack of family interaction, family conflicts, parental SA, and parental neglect [23, 49, 50]. Li *et al*. emphasized the effect of the interaction between family functioning and the capacity of undergraduates to be alone on SA [51].

The association between smartphone overuse and psychological manifestations has been observed shortly after the spread of the use of smartphones; the term nomophobia, which stands for “no-mobile-phone-phobia,” was used in 2008 during a study conducted in the United Kingdom to describe mobile phone dependence [52]. SA has also been mentioned to later lead to multiple psychological problems, such as sleep disorders, anxiety, depression, and suicidal behaviors [7, 8, 37, 53, 54]. K-6 score is widely used for assessing the presence of common mental health conditions, such as anxiety and depressive disorders, among diverse populations [20, 55]. Consistent with the studies mentioned above, 40% of participants suffered from psychological distress and 82.1% of them were smartphone addicts. In addition, the study has revealed the increase in daily screen time to be correlated with an increase in K-6 scores. Moreover, direct inquiry about complaining of depression showed 79.2% of smartphone addicts to be depressed, which has already been proven in multiple previous studies [18, 56, 57].

The classification of smartphone overuse as an addiction does not rely only on psychological studies; Liu *et al*. concluded dopamine to have an important role in the development of internet addiction [58]. Emerging evidence indicates SA to be associated with decreased levels and function of dopamine [59]. The same findings have been described before in other types of addiction [60]. Moreover, another study using functional Magnetic Resonance Imaging (MRI) described the similarities of activated brain regions between smartphone addicts and drug addicts [61]. Decreased gray matter in the brain cortex among smartphone addicts has also been reported [17].

The association between SA and psychiatric disorders is not fully understood; such disorders are frequently seen in the literature as results of SA, and the role of already existing psychiatric disorders as underlying risk factors for developing SA has not been widely investigated. Specific characteristics observed among smartphone addicts, such as their personality profiles and some psychological dimensions, could be risk factors for internet addiction disorder [47]. Matar Boumosleh *et al*. concluded undergraduates with personality type A along with increased distress and low mood as highly susceptible to developing SA [62].

Among the growing number of physical health disorders that affect physical well-being linked to SA is the Text Neck Syndrome (TNS), which is an emerging disorder in the last few decades associated with SA and characterized by cervical spinal degeneration and increased neck disability index due to abnormal head position during prolonged smartphone use [16, 63-66]. Impaired proprioception and wrong posture among smartphone addicts play a critical role in developing TNS and other musculoskeletal problems [67, 68].

Our findings have been found to be in agreement with previous studies on neck problems; the association between SA and neck pain has been reported to be highly significant. In addition to neck pain, our study findings have revealed complaints of pain in shoulder girdles, lower arms, and upper back to have a statistically significant association with SA. This association has been proven in previous studies indicating the strong relation between SA and musculoskeletal symptoms in different upper body areas [10, 69-71].

SA has also been reported as associated with pain in the lower back, right hip, and right leg (Table **4**), being in line with another study that has revealed a significant association between SA and musculoskeletal complaints in the lower back, hip, and feet [11]. Interestingly, significant right-side musculoskeletal pain among smartphone addicts, proven in our study, has also been observed before [11]. In addition, a weaker handgrip resulting from prolonged smartphone use, especially in the dominant hand, has been reported [62, 72]. This finding may be linked to the overuse of the dominant body part among smartphone addicts.

The study findings have also shown a significant association between SA and fatigability, as observed in previous studies; increased boredom, fatigue, exhaustion, and frustration have been reportedly linked to SA [73, 74].

In addition, the study findings have supported the previously revealed association between SA and headache [75, 76]. Sleep disturbance, poor sleep quality, insomnia, and sleep insufficiency have been previously described as SA-associated problems [7, 77-79]. Our findings have also shown a strong association between sleep disturbance and SA (*P* < .000). Furthermore, our study has revealed SA to be associated with cognitive symptoms, being in line with previous studies related to SA, and shown cognitive abnormalities, such as impaired memory, concentration difficulties, and bradyphrenia that can lead to lower cognitive abilities and unsatisfactory academic performance [11, 80, 81].

After around three decades since the first description of internet addiction, we believe that the time is coming to put all proven psychological and physical manifestations together to shape a new clinical identity with particular addictive, psychological, and physical components; this might help to develop a holistic approach to a specific clinical syndrome, namely “Smart Device Syndrome (SDS).” The primary components of the suggested syndrome are illustrated in Table **5** and need further prospective studies for refining and clinical validation.

Finally, this study has involved some inherent limitations related to applying a self-administered questionnaire, such as the possibility of information and recall bias. In addition, most of the participants were Syrian young adults, precluding the generalization of the results to other age groups or populations.

## CONCLUSION

This study has provided preliminary information related to SA among Syrian undergraduates and revealed a high prevalence of SA with a broad spectrum of associated mental and physical manifestations.

Moreover, the SA-proven psychological and physical manifestations can be combined together to shape a new clinical identity for developing a holistic approach to managing such disorders associated with a delineated clinical syndrome, namely “Smart Device Syndrome.” Further studies are needed to investigate the pathological mechanisms of SA and its associations.

## Figures and Tables

**Appendix 1:** English version of SAS-SV.

**Items**		**Strongly Disagree**	**Disagree**	**Weakly Disagree**	**Weakly Agree**	**Agree**	**Strongly Agree**
1	Missing planned work due to smartphone use	1	2	3	4	5	6
2	Having a hard time concentrating in class, while doing assignments, or while working due to smartphone use	1	2	3	4	5	6
3	Feeling pain in the wrists or at the back of the neck while using a smartphone	1	2	3	4	5	6
4	Won’t be able to stand not having a smartphone	1	2	3	4	5	6
5	Feeling impatient and fretful when I am not holding my smartphone	1	2	3	4	5	6
6	Having my smartphone in my mind even when I am not using it	1	2	3	4	5	6
7	I will never give up using my smartphone even when my daily life is already greatly affected by it.	1	2	3	4	5	6
8	Constantly checking my smartphone so as not to miss conversations between other people on Twitter or Facebook	1	2	3	4	5	6
9	Using my smartphone longer than I had intended	1	2	3	4	5	6
10	The people around me tell me that I use my smartphone too much.	1	2	3	4	5	6

**Note: **Kwon M, Kim DJ, Cho H, Yang S. The smartphone addiction scale: development and validation of a short version for adolescents. PLoS One. 2013 Dec 31;8(12):e83558. doi: 10.1371/journal.pone.0083558. PMID: 24391787; PMCID: PMC3877074.

**Table:** The Kessler Psychological Distress Scale (K-6). Kessler's instrument asks how often, during the past 30 days, the respondent felt:

-	**None of the Time**	**A Little of the Time**	**Some of the Time**	**Most of the Time**	**All the Time**
So sad that nothing could cheer you up?	0	1	2	3	4
Nervous?	0	1	2	3	4
Restless or fidgety?	0	1	2	3	4
Hopeless?	0	1	2	3	4
Doing everything was an effort?	0	1	2	3	4
Worthless?	0	1	2	3	4

**Note: **KESSLER, R., ANDREWS, G., COLPE, L., HIRIPI, E., MROCZEK, D., NORMAND, S., . . . ZASLAVSKY, A. (2002). Short screening scales to monitor population prevalence and trends in non-specific psychological distress. Psychological Medicine, 32(6), 959-976. doi:10.1017/S0033291702006074

**Table 1:** Participants’ demographic data (N, %).

-	***n***	**(%)**
**Gender**	-	-
Male	565	(40.3)
Female	836	(59.7)
**Age**	-	-
≤ 20	430	(30.7)
21-23	596	(42.5)
24-26	233	(16.6)
27-29	89	(6.4)
≥ 30	53	(3.8)
**Marital status**	-	-
Single	1314	(83.8)
Married	87	(6.2)
**Family size**	-	-
≤ 5	427	(30.5)
> 5	974	(69.5)
**Economic status**	-	-
Weak	147	(10.5)
Average	835	(59.6)
Good	369	(26.3)
Very good	50	(3.6)

**Table 2:** Smartphone usage data, including the purposes of smartphone use (more than one purpose accepted).

-	***n***	**(%)**
**Years of smartphone ownership**	-	-
*≤* 4	207	(14.8)
5–8	728	(52.0)
*≥* 9	466	(33.2)
**Mean daily use time of smartphone**	-	-
<4	441	(31.5)
4- <7	398	(28.4)
7- <10	303	(21.6)
≥ 10	259	(18.5)
**The purposes of smartphone use**	-	-
Social communication	1272	(90.2)
Playing	500	(35.7)
Learning	916	(65.4)
Internet surfing	1133	(80.9)
Online work	184	(13.1)
Shopping	125	(8.9)

**Table 3:** Binary logistic regression analysis of age group, mean daily screen time, and academic majors in relation to SA (N =1401).

**Variables**	**B**	**S.E.**	**Wald Test**	***P*-value**	**OR**
**Age group**					
18-20	.817	.319	6.544	0.011*	2.264
21-23	.744	.314	5.596	0.018*	2.104
24-26	.505	.329	2.361	0.124	1.657
27-29	.421	.375	1.262	0.261	1.523
≥ 30	Ref.	Ref.	Ref.	Ref.	Ref.
**Mean daily screen time (hours)**					
<4	-2.508	.293	73.383	0.000***	.081
4- <7	-1.079	.155	48.616	0.000***	.340
7- <10	-.609	.170	12.891	0.000***	.544
≥ 10	Ref.	Ref.	Ref.	Ref.	Ref.
**Academic major**					
Medical sciences	.775	.259	8.935	0.003**	2.170
Information and computer science	.579	.295	3.838	0.050	1.784
Sciences	.778	.376	4.285	0.038*	2.178
Literature and human	.542	.300	3.266	0.071	1.719
Engineering	.543	.275	3.903	0.048*	1.721
Faculty of education	.246	.354	.485	0.486	1.279
Law	.305	.355	.739	0.390	1.356
Economics	.711	.322	4.861	0.027*	2.035
Media	.211	.399	.280	0.597	1.235
Applied sciences	Ref.	Ref.	Ref.	Ref.	Ref.

**Note: **B = estimated parameter (effect); SE = standard error; OR = odds ratio.

Age group ≥ 30, mean daily time of smartphone use (hours) ≥10, and applied science were used as reference categories in the estimated logistic regression model (*P < 0.05, **P < 0.01, ***P <0.001).

**Table 4:** The statistical association between SA and relevant clinical presentations.

-	**Non-smartphone-addicted**		**Smartphone-addicted**		***P*-value**	**Z-test**
	***n***	**(%)**	***n***	**(%)**	-	-
**Pain area**						
Chest	43	(9.5)	111	(11.7)	0.23014	1.2019
Abdomen	178	(39.5)	437	(46.0)	<0.0001***	3.4826
L. jaw	21	(4.7)	50	(5.3)	0.6285	0.4838
L. shoulder girdle	55	(12.2)	176	(18.5)	0.0028**	2.9837
L. upper arm	26	(5.8)	121	(12.7)	<0.0001***	3.9784
L. lower arm	22	(4.9)	79	(8.3)	0.02034*	2.3244
R. jaw	10	(2.2)	54	(5.7)	0.00374**	2.9037
R. shoulder girdle	51	(11.3)	195	(20.5)	<0.0001***	4.2369
R. upper arm	31	(6.9)	142	(14.9)	<0.0001***	4.2916
R. lower arm	25	(5.5)	113	(11.9)	0.0002***	3.7274
L. hip	34	(7.5)	87	(9.2)	0.3125	1.008
L. upper leg	27	(6.0)	68	(7.2)	0.41794	0.8146
L. lower leg	24	(5.3)	59	(6.2)	0.50926	0.6585
R. hip	21	(4.7)	109	(11.5)	<0.0001***	4.109
R. upper leg	19	(4.2)	84	(8.8)	0.00194**	3.1019
R. lower leg	15	(3.3)	87	(7.1)	<0.0001***	3.9252
Neck	161	(35.7)	496	(52.2)	<0.0001***	5.7863
Lower back	90	(20.0)	278	(29.3)	0.00022**	3.6985
Upper back	130	(28.8)	369	(38.8)	0.00026**	3.6582
**Other relevant symptoms**						
Headache	310	(68.7)	716	(75.4)	<0.0001***	20.4354
Fatigability	28	(6.2)	108	(11.4)	0.00228**	4.0479
Sleep disturbance	52	(11.5)	261	(27.5)	<0.0001***	6.6938
Cognitive symptoms	30	(6.7)	218	(17.7)	<0.0001***	7.4661
Depression	301	(66.7)	808	(79.2)	<0.0001***	5.6696

**Note: **L: left side; R: right side. The results are significant at *P < 0.05, **P < 0.01, and ***P < 0.001.

**Table 5:** Suggested smart device syndrome (SDS) components.

**Suggested Components**		Priority*
**Proven smart device addiction**		
By applying validated smartphone addiction scales, such as SAS-SV 2013		++++
**Clinical manifestations**		
**Psychological components**		
1	Psychological distress	++++
2	Anxiety	+++
3	Depression	+++
4	Social phobia	++
5	Sleep disturbance	++
6	Cognitive symptoms (impaired memory, concentration difficulties, or bradyphrenia)	+++
**Physical components**		
1	Headache	++++
2	Fatigability	+++
3	Neck pain (with/without cervical disc degeneration)	++++
4	Back pain	+++
5	Shoulder girdle pain	+++
6	Arm pain	+++
7	Abdominal pain	++
8	Pain in the dominant body side (jaw, shoulder girdle, arm, hip, and leg)	++++

Note: * The priorities were weighted according to the results of previous studies and recent study findings.

## Data Availability

The data supporting the findings of the article is available in the Zenodo Repository at [https://zenodo.org/ uploads/11239259], reference number [10.5281/zenodo.11239259].

## LIST OF ABBREVIATIONS

K-6: Kessler psychological distress scale
OR: Odds ratio
RR: Relative risk
SA: Smartphone addiction
SAS-SV 2013: Smartphone addiction scale-short version
SDS: Smart device syndrome
TNS: Text neck syndrome

## STANDARDS OF REPORTING

STROBE and SAGER guidelines were followed.
